# Supplementary material for: Germline modifiers of the tumor immune microenvironment implicate drivers of cancer risk and immunotherapy response
Source: Nat Commun. 2023 May 12;14:2744. doi: 10.1038/s41467-023-38271-5 (PMC10182072; doi:10.1038/s41467-023-38271-5)
Supplement: Supplementary file 1 — Supplementary Information [file 41467_2023_38271_MOESM1_ESM.pdf]

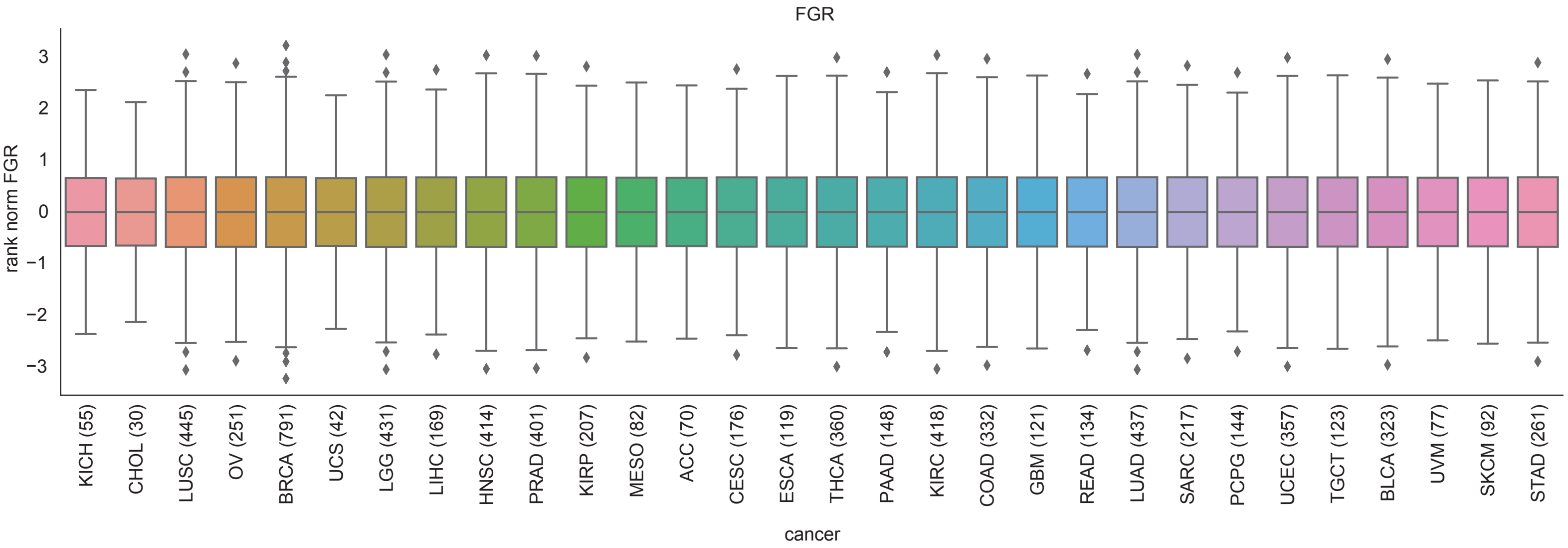


**Supplementary Figure 1: Identifying heritable characteristics of the tumor immune microenvironment, Related to Figure 1.** Boxplot of FGR expression values after inverse-rank normalization by TCGA[1] cancer type. Boxplots show median (line), 25th and 75th percentiles (box), and 1.5 × the interquartile range (IQR, whiskers).

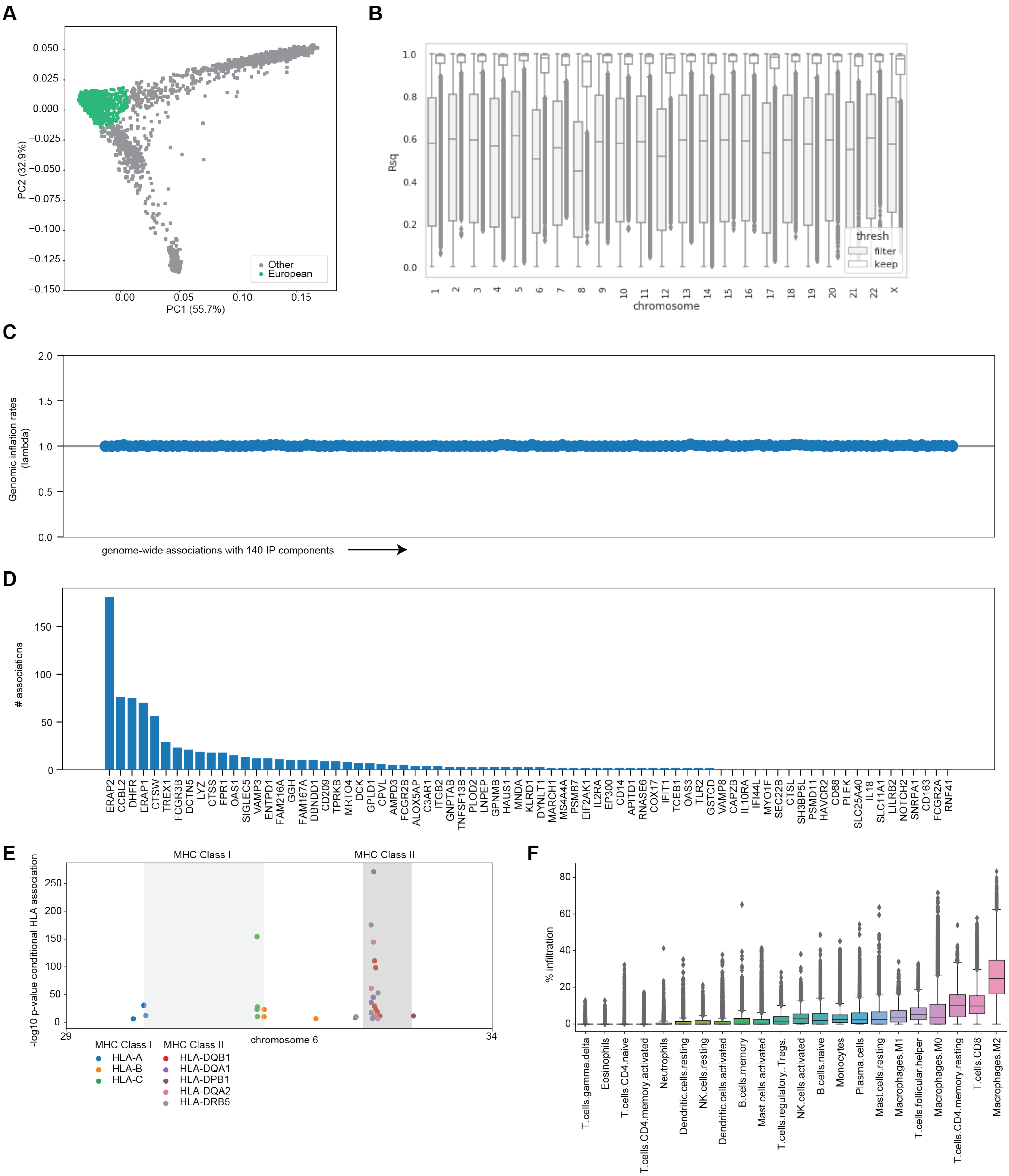
A
B
C
D
E
F
PC2 (32.9%)
PC1 (55.7%)
0.050
0.025
0.000
−0.025
−0.050
−0.075
−0.100
−0.125
−0.150
0.00
0.05
0.10
0.15
Other
European
Rsq
chromosome
1.0
0.8
0.6
0.4
0.2
0.0
1 2 3 4 5 6 7 8 9 10 11 12 13 14 15 16 17 18 19 20 21 22 X
thresh
filter
keep
Genomic inflation rates (lambda)
2.0
1.5
1.0
0.5
0.0
genome-wide associations with 140 IP components
# associations
150
100
50
0
ERAP2 CCBL2 DHFR ERAP1 CTSW TREX1 FCGR3B DCTN5 LYZ CTSS FPR1 OAS1 SIGLEC5 VAMP3 ENTPD1 FAM216A GGH FAM167A DBNDD1 CD209 TPRKB MRTO4 DCK GPLD1 CPVL AMPD3 FCGR2B ALOX5AP C3AR1 ITGB2 GNPTAB TNFSF13B PLOD2 LNPEP GPNMB HAUS1 MNDA KLRD1 DYNLT1 MARCH1 MS4A4A PSMB7 EIF2AK1 IL2RA EP300 CD14 APITD1 RNASE6 COX17 IFIT1 TCEB1 OAS3 TLR2 GSTCD VAMP8 CAPZB IL10RA IFI44L MYO1F SEC22B CTSL SH3BP5L PSMD11 HAVCR2 CD68 PLEK SLC25A40 IL18 SLC11A1 LILRB2 NOTCH2 SNRPA1 CD163 FCGR2A RNF41
-log10 p-value conditional HLA association
MHC Class I
MHC Class II
250
200
150
100
50
0
29
chromosome 6
34
MHC Class I
HLA-A
HLA-B
HLA-C
MHC Class II
HLA-DQB1
HLA-DQA1
HLA-DPB1
HLA-DQA2
HLA-DRB5
% infiltration
80
60
40
20
0
T.cells.gamma.delta
Eosinophils
T.cells.CD4.naive
T.cells.CD4.memory.activated
Neutrophils
Dendritic.cells.resting
NK.cells.resting
Dendritic.cells.activated
B.cells.memory
Mast.cells.activated
T.cells.regulatory..Tregs.
NK.cells.activated
B.cells.naive
Monocytes
Plasma.cells
Mast.cells.resting
Macrophages.M1
T.cells.follicular.helper
Macrophages.M0
T.cells.CD4.memory.resting
T.cells.CD8
Macrophages.M2

**Supplementary Figure 2: Detecting putative germline modifiers of the tumor immune microenvironment , Related to Figure 2. (A).** PCA analysis of TCGA[1] genotypes; green indicates European discovery cohort. **(B)** Boxplot of Rsq distribution of imputed genotypes across chromosomes. **(C)** Plot of inflation factors (lambda) for associations using PanCanAtlas data. **(D)** Barplot of number of associations for each immune phenotype (IP) components for 7357 TCGA European individuals. **(E)** Scatterplot of HLA associations based on chromosome 6 genomic location. **(F)** Boxplot of CIBERSORTx infiltration across TCGA individuals. Boxplots show median (line), 25th and 75th percentiles (box), and 1.5 × the interquartile range (IQR, whiskers).

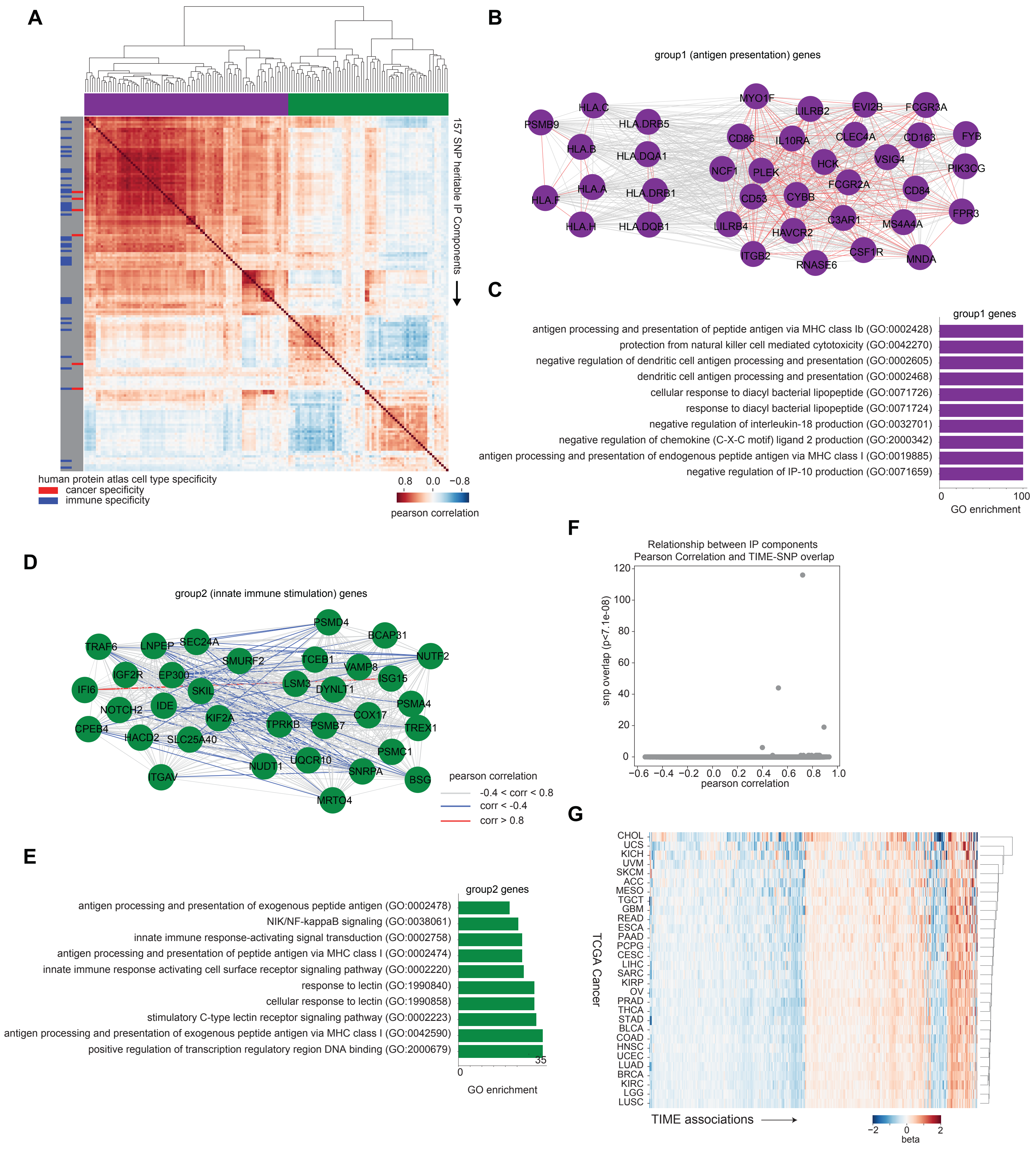
A
157 SNP heritable IP Components
human protein atlas cell type specificity
cancer specificity
immune specificity
0.8 0.0 −0.8
pearson correlation
B
group1 (antigen presentation) genes
HLA.C
PSMB9
HLA.DRB5
HLA.B
HLA.DQA1
HLA.A
HLA.DRB1
HLA.F
HLA.H
HLA.DQB1
MYO1F
LILRB2
EVI2B
FCGR3A
CD86
IL10RA
CLEC4A
CD163
FYB
NCF1
PLEK
HCK
VSIG4
PIK3CG
FCGR2A
CD53
CYBB
CD84
C3AR1
FPR3
LILRB4
HAVCR2
MS4A4A
ITGB2
CSF1R
RNASE6
MNDA
C
group1 genes
antigen processing and presentation of peptide antigen via MHC class Ib (GO:0002428)
protection from natural killer cell mediated cytotoxicity (GO:0042270)
negative regulation of dendritic cell antigen processing and presentation (GO:0002605)
dendritic cell antigen processing and presentation (GO:0002468)
cellular response to diacyl bacterial lipopeptide (GO:0071726)
response to diacyl bacterial lipopeptide (GO:0071724)
negative regulation of interleukin-18 production (GO:0032701)
negative regulation of chemokine (C-X-C motif) ligand 2 production (GO:2000342)
antigen processing and presentation of endogenous peptide antigen via MHC class I (GO:0019885)
negative regulation of IP-10 production (GO:0071659)
0 100
GO enrichment
D
group2 (innate immune stimulation) genes
PSMD4
BCAP31
TRAF6
LNPEP
SEC24A
NUTF2
SMURF2
TCEB1
VAMP8
IGF2R
EP300
ISG15
IFI6
LSM3
DYNLT1
SKIL
PSMA4
NOTCH2
IDE
COX17
KIF2A
CPEB4
TPRKB
PSMB7
TREX1
HACD2
SLC25A40
PSMC1
UQCR10
NUDT1
SNRPA
ITGAV
BSG
MRTO4
pearson correlation
-0.4 < corr < 0.8
corr < -0.4
corr > 0.8
E
group2 genes
antigen processing and presentation of exogenous peptide antigen (GO:0002478)
NIK/NF-kappaB signaling (GO:0038061)
innate immune response-activating signal transduction (GO:0002758)
antigen processing and presentation of peptide antigen via MHC class I (GO:0002474)
innate immune response activating cell surface receptor signaling pathway (GO:0002220)
response to lectin (GO:1990840)
cellular response to lectin (GO:1990858)
stimulatory C-type lectin receptor signaling pathway (GO:0002223)
antigen processing and presentation of exogenous peptide antigen via MHC class I (GO:0042590)
positive regulation of transcription regulatory region DNA binding (GO:2000679)
0 35
GO enrichment
F
Relationship between IP components
Pearson Correlation and TIME-SNP overlap
snp overlap (p<7.1e-08)
0 20 40 60 80 100 120
−0.6 −0.4 −0.2 0.0 0.2 0.4 0.6 0.8 1.0
pearson correlation
G
CHOL
UCS
KICH
UVM
SKCM
ACC
MESO
TGCT
GBM
READ
ESCA
PAAD
PCPG
CESC
LIHC
SARC
KIRP
OV
PRAD
THCA
STAD
BLCA
COAD
HNSC
UCEC
LUAD
BRCA
KIRC
LGG
LUSC
TCGA Cancer
TIME associations
−2 0 2
beta

**Supplementary Figure 3: Detecting putative germline modifiers of the tumor immune microenvironment, Related to Figure 2. (A)** Clustermap depicting 157 SNP-heritable IP components from two-state GCTA analysis and their pairwise correlation across 30 tumors in the TCGA[1]. **(B)** Network plot of group 1 (antigen presentation) genes from Pearson correlation clustermap analysis. **(C)** Top 10 GO enrichment terms and enrichment values from group 1 (antigen presentation) genes. **(D)** Network plot of group 2 (innate immune stimulation) genes from Pearson correlation Clustermap analysis. **(E)** Top 10 GO enrichment categories and enrichment values from group 2 (innate immune stimulation) genes. **(F)** Scatterplot of Pearson correlation of IP components and number of overlapping significant variants. **(G)** Clustermap of cancer type specific beta values for TIME eQTLs identified through pan-cancer analysis

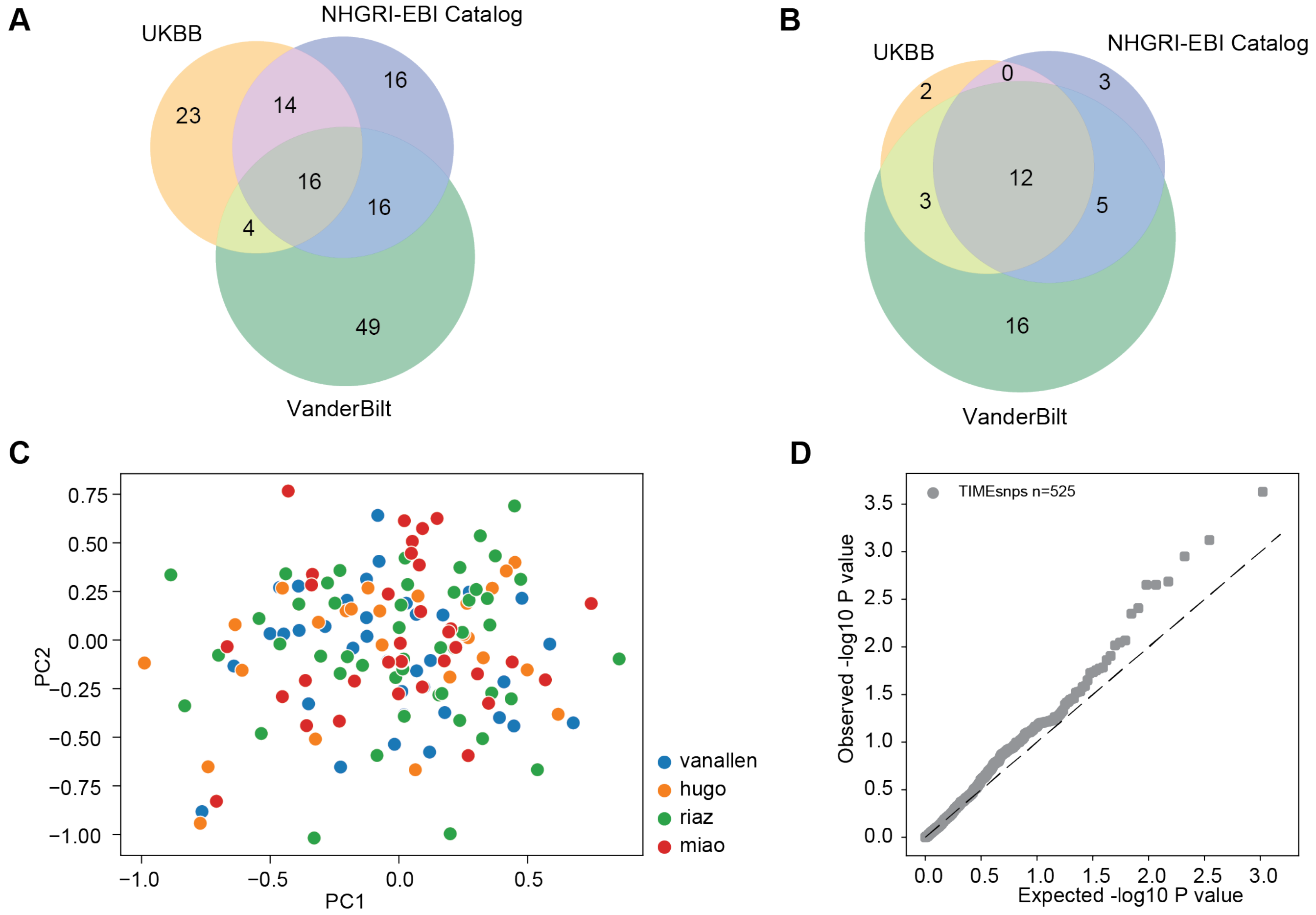


**Supplementary Figure 4: Identification of TIME eQTLs related to cancer outcomes (A)** Venn Diagram of overlap between TIME eQTLs implicated by UK Biobank[2] PheWAS, Vanderbilt PheWAS catalog and NHGRI-EBI GWAS Catalog (LDtrait). **(B)** Venn Diagram of overlap between genes implicated by TIME eQTLs by UK Biobank PheWAS, Vanderbilt PheWAS catalog and NHGRI-EBI GWAS Catalog. **(C)** PCA plot of TIME eQTLs implicated by polygenic ICB score (PICS). **(D)** Q-Q plot of METAL meta-analysis sample-size weighted association with immunotherapy response in Riaz *et al.*[3], Snyder *et al.*[4], Hugo *et al.*[5] and Van Allen *et al.*[6] melanoma discovery cohort.

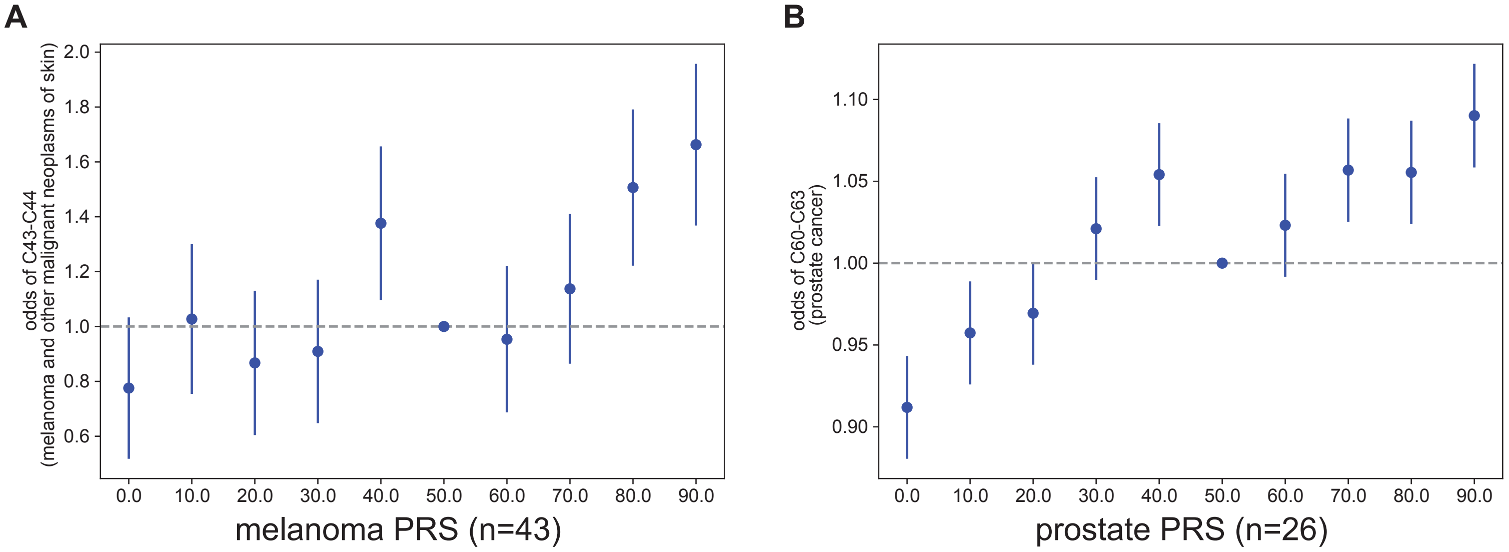


**Supplementary Figure 5: TIME eQTLs underlying antigen presentation stratify melanoma and prostate cancer risk. (A)** Logistic regression odds ratio +/- SE of melanoma risk among individuals in PRS (# snps = 43) quantiles in High Density Melanoma cohort[7,8] (n=1699). **(B)** Logistic regression odds ratio of prostate cancer risk among individuals in the top and bottom 10th quantile of PRS (# snps=26) in ELLIPSE consortium[9] (n=91,644). Error bars indicate 95% confidence interval.

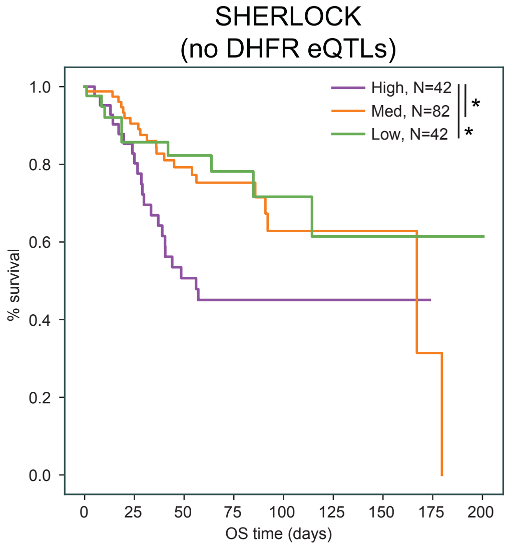


**Supplementary Figure 6: TIME eQTLs associated with survival implicate immune evasion, related to Figure 4.** Overall survival Kaplan-Meier curve based on LUAD PSS (excluding DHFR eQTLs) in SHERLOCK[10] (n=166). Significance is marked as: *p<.05, **p<.01, ***p<.001.

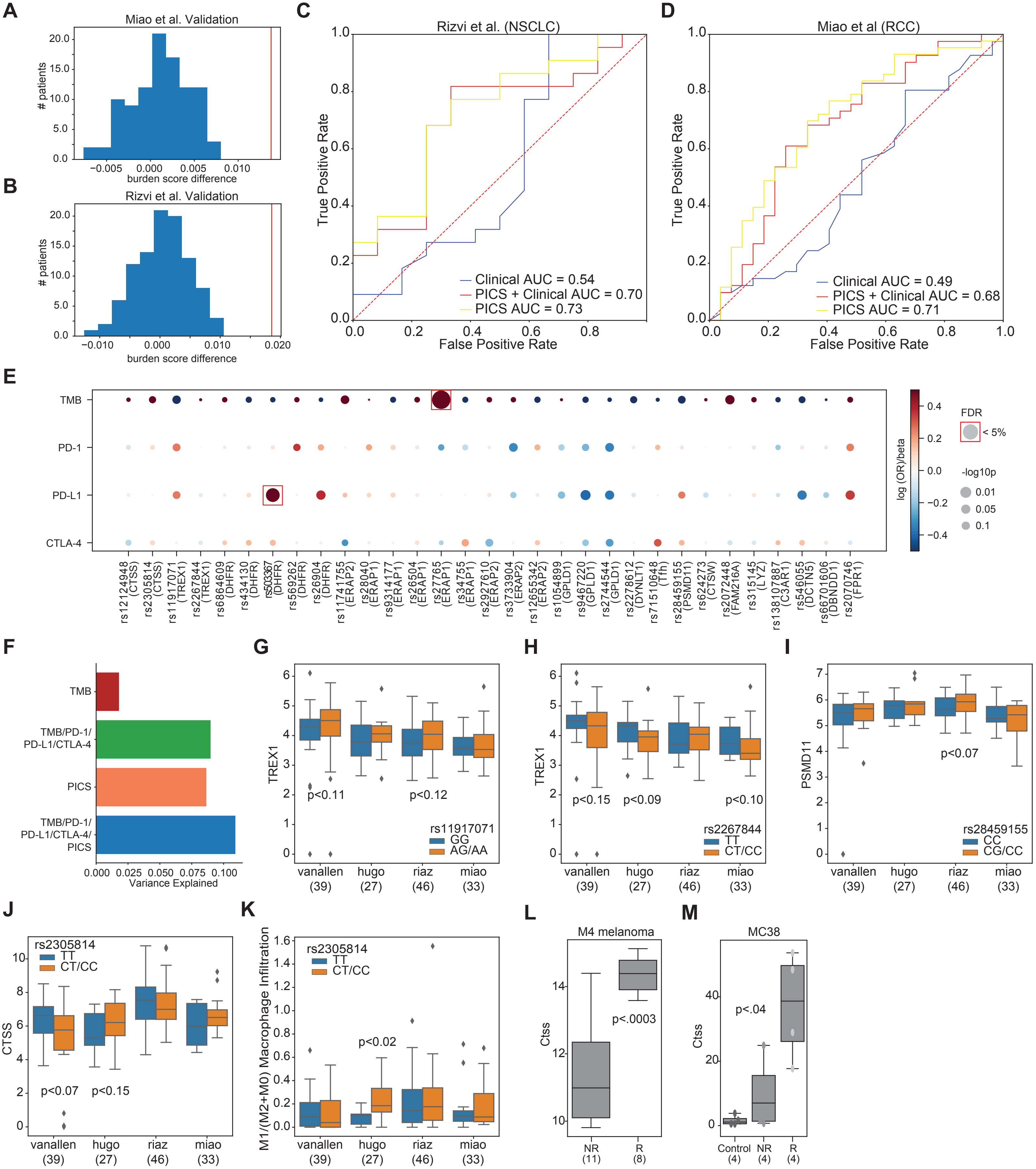
A
Miao et al. Validation
# patients
burden score difference
B
Rizvi et al. Validation
# patients
burden score difference
C
Rizvi et al. (NSCLC)
True Positive Rate
False Positive Rate
Clinical AUC = 0.54
PICS + Clinical AUC = 0.70
PICS AUC = 0.73
D
Miao et al (RCC)
True Positive Rate
False Positive Rate
Clinical AUC = 0.49
PICS + Clinical AUC = 0.68
PICS AUC = 0.71
E
TMB
PD-1
PD-L1
CTLA-4
log (OR)/beta
FDR
< 5%
-log10p
0.01
0.05
0.1
rs12124948 (CTSS)
rs2305814 (CTSS)
rs11917071 (TREX1)
rs2267844 (TREX1)
rs6864609 (DHFR)
rs434130 (DHFR)
rs503367 (DHFR)
rs569262 (DHFR)
rs26904 (DHFR)
rs11741755 (ERAP2)
rs28040 (ERAP1)
rs9314177 (ERAP1)
rs26504 (ERAP1)
rs27765 (ERAP1)
rs34755 (ERAP1)
rs2927610 (ERAP2)
rs3733904 (ERAP2)
rs12655342 (ERAP2)
rs1054899 (GPLD1)
rs9467220 (GPLD1)
rs2744544 (GPLD1)
rs2278612 (DYNLT1)
rs71510648 (Tfh)
rs28459155 (PSMD11)
rs624273 (CTSW)
rs2072448 (FAM216A)
rs315145 (LYZ)
rs13810787 (C3AR1)
rs546055 (DCTN5)
rs66701606 (DBNDD1)
rs2070746 (FPR1)
F
TMB
TMB/PD-1/ PD-L1/CTLA-4
PICS
TMB/PD-1/ PD-L1/CTLA-4/ PICS
Variance Explained
G
TREX1
p<0.11
p<0.12
rs11917071
GG
AG/AA
vanallen (39)
hugo (27)
riaz (46)
miao (33)
H
TREX1
p<0.15
p<0.09
p<0.10
rs2267844
TT
CT/CC
vanallen (39)
hugo (27)
riaz (46)
miao (33)
I
PSMD11
p<0.07
rs28459155
CC
CG/CC
vanallen (39)
hugo (27)
riaz (46)
miao (33)
J
CTSS
rs2305814
TT
CT/CC
p<0.07
p<0.15
vanallen (39)
hugo (27)
riaz (46)
miao (33)
K
M1/(M2+M0) Macrophage Infiltration
rs2305814
TT
CT/CC
p<0.02
vanallen (39)
hugo (27)
riaz (46)
miao (33)
L
M4 melanoma
Ctss
p<.0003
NR (11)
R (8)
M
MC38
Ctss
p<.04
Control (4)
NR (4)
R (4)

**Supplementary Figure 7: TIME eQTLs implicate targets for modulating immune responses, Related to Figure 5. (A)** Histogram of difference between polygenic ICB score (PICS) for 100 random bootstrapping trials compared to actual difference (red) in Miao *et al.*[11] validation cohort (n=70) **(B)** Histogram of difference PICS for 100 random bootstrapping trials compared to actual difference (red) in Rizvi *et al.*[12] validation cohort (n=34). **(C)** ROC-AUC analysis of PICS, clinical variables (age, sex) and PICS+clinical variables in Rizvi *et al.* validation cohort. **(D)** ROC-AUC analysis of PICS, clinical variables (age, sex) and PICS+clinical variables in Miao *et al.* validation cohort. **(E)** Grid plot of beta values of variant association with TMB, *CTLA-4, PD-1*, and *PD-L1* controlling for cohort. Unadjusted logistic regression p-values are indicated by dot size. Benjamini-Hochberg correction was used for false discovery rate (FDR) calculation. Associations passing FDR threshold 5% are outlined with red square. **(F)** Variance explained (pseudo-$R^2$) by TMB, TMB/PD1/PDL1/CTLA4, PICS and PICS/TMB/PD1/PDL1/CTLA4 in Miao *et al.* validation cohort. **(G)** Boxplot of rs11917071 association with *TREX1* expression across ICB cohorts. **(H)** Boxplot of rs2267844 association with *TREX1* expression across ICB cohorts. **(I)** Boxplot of rs28459155 association with *PSMD11* expression across ICB cohorts. **(J)** Boxplot of rs2305814 association with *CTSS* expression across ICB cohorts. **(K)** Boxplot of rs2305814 association with M1/M2+M0 macrophage infiltration across ICB cohorts. **(L)** *Ctss* expression in non-responder and responder of M4 melanoma mouse model. Sample sizes for each cohort are indicated in parentheses. **(M)** *Ctss* expression in non-responder and responder of MC38 mouse model. Boxplots show median (line), 25th and 75th percentiles (box), and 1.5 × the interquartile range (IQR, whiskers). Two-sided Mann-Whitney U p-values were used for comparisons, adjusted if > 2 comparisons were being made.

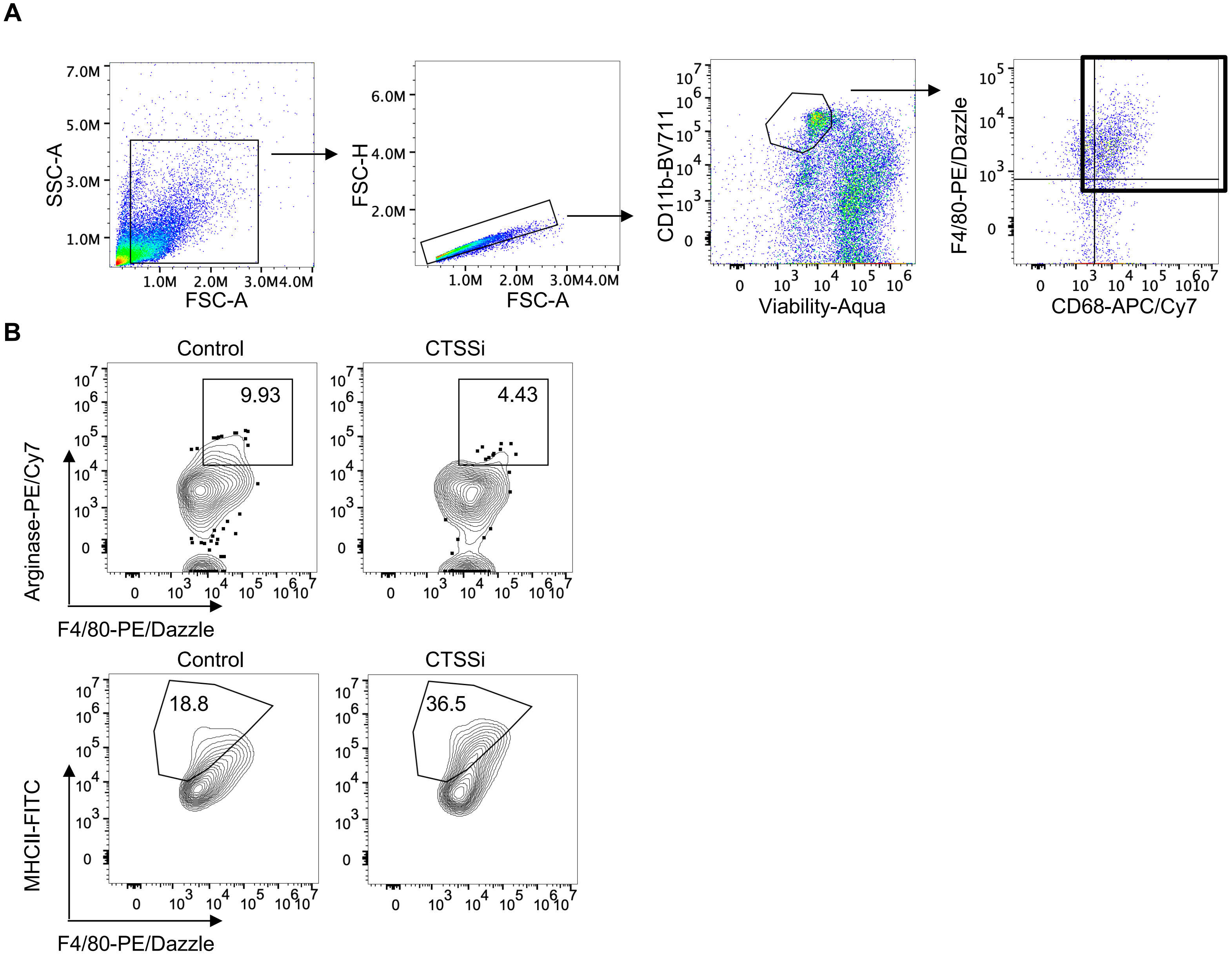


**Supplementary Figure 8: Flow cytometry gating strategy for MC38 CTSSi experiments, Related to Figure 5.** **(A)** Single cells were gated by FSC-A vs FSC-H, then live CD11b cells were gated, and macrophages were characterized by CD68+ and F4/80+. **(B)** Representative flow cytometry plot of tumor-infiltrating M1 macrophages (F4/80+ MHCII+) and M2 macrophages (F4/80+ Arginase+) following CTSSi treatment.

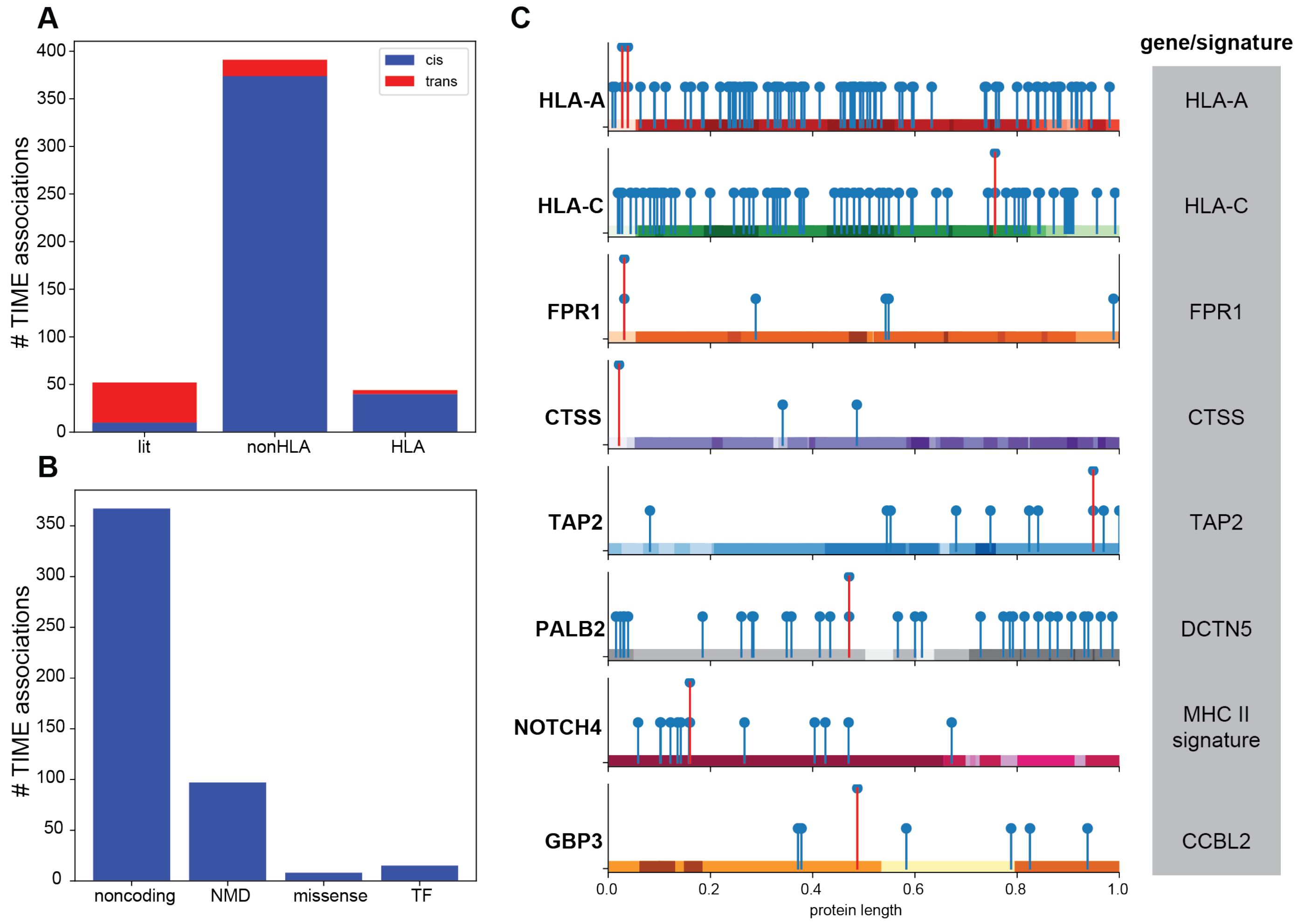


**Supplementary Figure 9: Characterization of genes implicated by PICS model TIME eQTLs, Related to Figure 7. (A)** Barplot quantifying the number of cancer relevant TIME eQTLs which are *trans* (>1 MB from the TSS of the associated IP component) and *cis.* **(B)**. Barplot describing the effects of cancer relevant TIME eQTLs **(C)** Location of missense TIME eQTLs (red) and known genetic variation (blue) in the coding sequence of affected genes. Associated gene on the right side (gray).

## Supplementary References


1. Hoadley, K. A. *et al.* Cell-of-Origin Patterns Dominate the Molecular Classification of 10,000 Tumors from 33 Types of Cancer. *Cell* **173**, 291–304.e6 (2018).

2. Bycroft, C. *et al.* The UK Biobank resource with deep phenotyping and genomic data. *Nature* **562**, 203–209 (2018).

3. Riaz, N. *et al.* Tumor and Microenvironment Evolution during Immunotherapy with Nivolumab. *Cell* **171**, 934–949.e16 (2017).

4. Snyder, A. *et al.* Genetic basis for clinical response to CTLA-4 blockade in melanoma. *N. Engl. J. Med.* **371**, 2189–2199 (2014).

5. Hugo, W. *et al.* Genomic and Transcriptomic Features of Response to Anti-PD-1 Therapy in Metastatic Melanoma. *Cell* **168**, 542 (2017).

6. Van Allen, E. M. *et al.* Genomic correlates of response to CTLA-4 blockade in metastatic melanoma. *Science* **350**, 207–211 (2015).

7. Li, C. *et al.* Genetic variants and haplotypes of the caspase-8 and caspase-10 genes contribute to susceptibility to cutaneous melanoma. *Hum. Mutat.* **29**, 1443–1451 (2008).

8. Li, C. *et al.* Haplotype and genotypes of the VDR gene and cutaneous melanoma risk in non-Hispanic whites in Texas: a case-control study. *Int. J. Cancer* **122**, 2077–2084 (2008).

9. Conti, D. V. *et al.* Trans-ancestry genome-wide association meta-analysis of prostate cancer identifies new susceptibility loci and informs genetic risk prediction. *Nat. Genet.* **53**, 65–75 (2021).

10. Zhang, T. *et al.* Genomic and evolutionary classification of lung cancer in never smokers. *Nat. Genet.* **53**, 1348–1359 (2021).

11. Miao, D. *et al.* Genomic correlates of response to immune checkpoint therapies in clear cell renal cell carcinoma. *Science* **359**, 801–806 (2018).

12. Rizvi, N. A. *et al.* Cancer immunology. Mutational landscape determines sensitivity to PD-1 blockade in non-small cell lung cancer. *Science* **348**, 124–128 (2015).
