# Supplementary material for: Germline modifiers of the tumor immune microenvironment implicate drivers of cancer risk and immunotherapy response
Source: Nat Commun. 2023 May 12;14:2744. doi: 10.1038/s41467-023-38271-5 (PMC10182072; doi:10.1038/s41467-023-38271-5)
Supplement: Supplementary file 3 — Description of Additional Supplementary Files [file 41467_2023_38271_MOESM3_ESM.pdf]

Description of Additional Supplementary Files

File Name: Supplementary Data 1
Description: TCGA Cancer type abbreviations and sample counts

File Name: Supplementary Data 2
Description: Description of IP Components

File Name: Supplementary Data 3
Description: TCGA IP component within cancer type rank-normalized values

File Name: Supplementary Data 4
Description: Genome-wide Complex Trait Analysis (GCTA) results for full TCGA

File Name: Supplementary Data 5
Description: Genome-wide Complex Trait Analysis (GCTA) results for BRCA

File Name: Supplementary Data 6
Description: Significant TIME Associations from GWAS Analysis of 157 IP Components

File Name: Supplementary Data 7
Description: Significant TIME Associations from conditional GWAS Analysis of HLA-DRB5 using only individuals with HLA-DRB1*15 and HLA-DRB1*16 alleles

File Name: Supplementary Data 8
Description: IP components contributing most to loadings for the first 10 PCs from PCA analysis of 157 IP components across TCGA samples

File Name: Supplementary Data 9
Description: TIME eQTL association with cancer type

File Name: Supplementary Data 10
Description: Literature SNP Significant TIME Associations

File Name: Supplementary Data 11
Description: UKBioBank PheWAS Results for TIME eQTLs

File Name: Supplementary Data 12
Description: Kaplan-Meier Survival Analysis of TIME eQTLs

File Name: Supplementary Data 13
Description: Covariates included for CoxPH survival analysis by tumor type

File Name: Supplementary Data 14
Description: TCGA exome-imputed versus SNP Array measured mismatch frequency

File Name: Supplementary Data 15
Description: METAL Immune-Checkpoint Blockade (ICB) association analysis results

File Name: Supplementary Data 16
Description: Association of TIME eQTLs and Immune-Checkpoint Blockade (ICB) markers with ICB Response

File Name: Supplementary Data 17
Description: DESeq2 analysis of ICB variant TIME genes

File Name: Supplementary Data 18
Description: Selection criteria for prioritizing ICB TIME genes for validation studies

File Name: Supplementary Data 19
Description: CTSS primer sequences

File Name: Supplementary Data 20
Description: GREGOR histone mark enrichment analysis of TIME eQTLs

File Name: Supplementary Data 21
Description: Significant cell-type associations of TIME eQTLs
