# Supplementary material for: Germline modifiers of the tumor immune microenvironment implicate drivers of cancer risk and immunotherapy response
Source: Nat Commun. 2023 May 12;14:2744. doi: 10.1038/s41467-023-38271-5 (PMC10182072; doi:10.1038/s41467-023-38271-5)
Supplement: Supplementary file 5 — Reporting Summary [file 41467_2023_38271_MOESM5_ESM.pdf]

nature portfolio

Corresponding author(s): Hannah Carter

Last updated by author(s): 04/19/2023

Reporting Summary

Nature Portfolio wishes to improve the reproducibility of the work that we publish. This form provides structure for consistency and transparency in reporting. For further information on Nature Portfolio policies, see our Editorial Policies and the Editorial Policy Checklist.

Statistics

For all statistical analyses, confirm that the following items are present in the figure legend, table legend, main text, or Methods section.

n/a	Confirmed	
☐	☒	The exact sample size (*n*) for each experimental group/condition, given as a discrete number and unit of measurement
☐	☒	A statement on whether measurements were taken from distinct samples or whether the same sample was measured repeatedly
☐	☒	The statistical test(s) used AND whether they are one- or two-sided<br>*Only common tests should be described solely by name; describe more complex techniques in the Methods section.*
☐	☒	A description of all covariates tested
☐	☒	A description of any assumptions or corrections, such as tests of normality and adjustment for multiple comparisons
☐	☒	A full description of the statistical parameters including central tendency (e.g. means) or other basic estimates (e.g. regression coefficient) AND variation (e.g. standard deviation) or associated estimates of uncertainty (e.g. confidence intervals)
☐	☒	For null hypothesis testing, the test statistic (e.g. *F*, *t*, *r*) with confidence intervals, effect sizes, degrees of freedom and *P* value noted<br>*Give P values as exact values whenever suitable.*
☒	☐	For Bayesian analysis, information on the choice of priors and Markov chain Monte Carlo settings
☒	☐	For hierarchical and complex designs, identification of the appropriate level for tests and full reporting of outcomes
☐	☒	Estimates of effect sizes (e.g. Cohen's *d*, Pearson's *r*), indicating how they were calculated

Our web collection on statistics for biologists contains articles on many of the points above.

Software and code

Policy information about availability of computer code

Data collection	TCGA data was downloaded via the gdc-client (v1.6.0). dbGaP data was downloaded using ascp from Aspera Connect (v3.9.1.168302) and the SRA toolkit (v2.9.6-1-ubuntu64).
Data analysis	Genotype processing of TCGA data was conducted with BIRDSUITE (RRID: SCR_001794) and PLINK (RRID:SCR_001757). Genotypes were imputed with Michigan Imputation Server (RRID: SCR_017579), reference panel: HRC Version r1.1 2016 with Eaglev2.3 phasing. Genetic ancestry inference was conducted with sklearn v0.20.3 k-means clustering and aberrant R package v1.0 using HAPMAP Phase III genotypes lifted over using liftOver (downloaded 07-09-2019). SNP-heritability analysis was conducted with Genome-wide Complex Trait Analysis (GCTA) v1.93.2beta. Principal component analysis (PCA) was conducted with sklearn v0.20.3. GWAS was conducted with PLINK (RRID:SCR_001757). Cancer risk analysis involved analysis of Vanderbilt PheWAS catalog, NHGRI-EBI GWAS catalog and UK Biobank using PLATO v2.0.0. Statistical analysis and multiple hypothesis testing correction were conducted with statsmodels and scipy v1.2.1. Survival analysis was conducted with lifelines v0.25.11 package. Epigenetic analysis was conducted with GREGOR (RRID: SCR_009165) on 479 bed files for 11 histone experiments and 52 cell types from ENCODE (RRID:SCR_015482). DICE eQTLs were obtained at https://dice-database.org/. For immune checkpoint blockade analysis, raw fastq files were processed with BWA (RRID:SCR_010910) v0.7.17-r1188, sorted by SAMTOOLS (RRID:SCR_002105) v0.1.19, marked for duplicates with Picard Tools (RRID:SCR_006525) v2.12.3 and recalibrated with GATK (RRID:SCR_001876) v3.8-1-0. Germline variants were called from sorted BAM files using DeepVariant v0.10.0-gpu. For RNA-seq processing, FASTQ/BAM files were downloaded. BAM files were converted to FASTQ using bam2fq. Unpaired reads were removed using fastq pair. Paired reads were aligned with STAR (RRID:SCR_004463) v2.4.1d to GRCh37 reference alignment. RSEM v1.2.21 was used for transcript quantification. Association with responder status was conducted with PLINK (RRID:SCR_001757) and METAL, version released 2011-03-25, using a sample size weighting scheme. To replicate analysis, code is available at https://github.com/cartercompbio/TIMEgermline.

For manuscripts utilizing custom algorithms or software that are central to the research but not yet described in published literature, software must be made available to editors and reviewers. We strongly encourage code deposition in a community repository (e.g. GitHub). See the Nature Portfolio guidelines for submitting code & software for further information.

Data

Policy information about availability of data

All manuscripts must include a data availability statement. This statement should provide the following information, where applicable:

- Accession codes, unique identifiers, or web links for publicly available datasets
- A description of any restrictions on data availability
- For clinical datasets or third party data, please ensure that the statement adheres to our policy

The data that support the findings of this study are available on request from the corresponding authors H.C. and M.P. The data are not publicly available as they were accessed through dbGaP applications and are controlled data that could lead to reidentification of individuals. For Data Access to processed genotyping, transcriptomic and mutation data, contact corresponding authors with proof of access to dbGaP studies: TCGA (dbgap accession: phs000178.v11.p8); UK Biobank (project ID 37671); Hugo et al. melanoma 2016 (SRA accession: SRP090294, SRP067938); Van Allen et al. melanoma 2015 (dbgap accession: phs000452.v3.p1, SRA accession: SRP011540); Miao et al. renal cell carcinoma 2018 (dbgap accession: phs001493.v2.p1, SRA accession: SRP128156, Cancer); Riaz et al. melanoma 2017 (SRA accession: SRP095809, SRP094781); Rizvi et al. non-small cell lung carcinoma 2015 (dbgap accession: phs000980.v1.p1, SRA accession: SRP064805); Snyder et al. melanoma 2014 (dbgap accession: phs001041.v1.p1, SRA accession: SRP072934); Oncoarray Prostate Cancer (dbgap accession: phs001391.v1.p1); Melanoma Exome Sequencing (dbgap accession: phs000933.v3.p1).

Human research participants

Policy information about studies involving human research participants and Sex and Gender in Research.

Reporting on sex and gender	Both females and male sex participants were included in analyses, and sex was included as covariate in discovery and validation analyses. Findings are applicable to both sexes.
Population characteristics	*Describe the covariate-relevant population characteristics of the human research participants (e.g. age, genotypic information, past and current diagnosis and treatment categories). If you filled out the behavioural & social sciences study design questions and have nothing to add here, write "See above."*
Recruitment	TCGA participants enrolled at TCGA Tissue Source Sites where biospecimens and clinical data were collected in accordance with HIPAA policies.
Ethics oversight	*Identify the organization(s) that approved the study protocol.*

Note that full information on the approval of the study protocol must also be provided in the manuscript.

Field-specific reporting

Please select the one below that is the best fit for your research. If you are not sure, read the appropriate sections before making your selection.

☒ Life sciences ☐ Behavioural & social sciences ☐ Ecological, evolutionary & environmental sciences

For a reference copy of the document with all sections, see nature.com/documents/nr-reporting-summary-flat.pdf

Life sciences study design

All studies must disclose on these points even when the disclosure is negative.

Sample size	This study required matched genotype and phenotype information from publicly available datasets, which was the limiting factor on sample size. TCGA was selected for discovery analysis as it is the largest dataset that provides genotype and phenotype information with tumor samples profiled for 11,542 samples from 10,875 unique individuals. UK Biobank was used for cancer risk assessment and included genotype and ICD10 code information for ~487,000 individuals. Validation cohorts for cancer risk assessment in melanoma and prostate cancer include GENEVA High-Density Melanoma cohort with 3033 individuals and Oncoarray Prostate Cancer cohort with 91,644 individuals. For immune checkpoint blockade response assessment, genotype and treatment response information for 276 melanoma, 68 renal cell carcinoma and 34 non-small cell lung cancer patients treated with immune checkpoint blockade (anti-PD-1, anti-CTLA4, combo) were analyzed.
Data exclusions	Data exclusion criteria were pre-established. For discovery cohort analysis, immune-cell derived cancers were excluded (DLBC, LAML, and THYM). Related individuals were excluded (KING kinship coefficient > 0.177, which represents first-degree relatedness). Quality control exclusion criteria for individuals excluded individuals with genotype coverage <90% and high heterozygosity rates (>3 SDs of mean). TCGA cancer types (TGCT,PCPG) which did not meet quality standards per Liu et al were excluded from survival analysis as survival data did not meet quality standards. Non-European individuals were excluded from TCGA and UK Biobank analyses.
Replication	Polygenic risk, survival and ICB scores were all validated. Polygenic risk scores were constructed using UK Biobank melanoma and prostate cancer cohorts and validated in Geneva melanoma cohort and ELLIPSE prostate cancer cohort, respectively. Polygenic survival scores were validated using 70:30 split partitions. TCGA LUAD polygenic survival score was validated in Sherlock cohort. Polygenic ICB scores were constructed from four published melanoma cohorts and validated in non-small cell lung cancer and renal cell carcinoma cohort.

Randomization	Randomization was not applicable to our study. The current study did not involve an intervention (there was no random assignment of samples to groups prior to data collection or analysis), and authors had no input on how samples were included in the TCGA, UK Biobank and immune checkpoint blockade (ICB) cohorts. Bootstrapping for ICB response analysis was conducted and described in more detail in methods.
Blinding	Blinding was not applicable as data collection was not performed in study. Other than exclusion criteria described above, all data was analyzed and presented.

Behavioural & social sciences study design

All studies must disclose on these points even when the disclosure is negative.

Study description	*Briefly describe the study type including whether data are quantitative, qualitative, or mixed-methods (e.g. qualitative cross-sectional, quantitative experimental, mixed-methods case study).*
Research sample	*State the research sample (e.g. Harvard university undergraduates, villagers in rural India) and provide relevant demographic information (e.g. age, sex) and indicate whether the sample is representative. Provide a rationale for the study sample chosen. For studies involving existing datasets, please describe the dataset and source.*
Sampling strategy	*Describe the sampling procedure (e.g. random, snowball, stratified, convenience). Describe the statistical methods that were used to predetermine sample size OR if no sample-size calculation was performed, describe how sample sizes were chosen and provide a rationale for why these sample sizes are sufficient. For qualitative data, please indicate whether data saturation was considered, and what criteria were used to decide that no further sampling was needed.*
Data collection	*Provide details about the data collection procedure, including the instruments or devices used to record the data (e.g. pen and paper, computer, eye tracker, video or audio equipment) whether anyone was present besides the participant(s) and the researcher, and whether the researcher was blind to experimental condition and/or the study hypothesis during data collection.*
Timing	*Indicate the start and stop dates of data collection. If there is a gap between collection periods, state the dates for each sample cohort.*
Data exclusions	*If no data were excluded from the analyses, state so OR if data were excluded, provide the exact number of exclusions and the rationale behind them, indicating whether exclusion criteria were pre-established.*
Non-participation	*State how many participants dropped out/declined participation and the reason(s) given OR provide response rate OR state that no participants dropped out/declined participation.*
Randomization	*If participants were not allocated into experimental groups, state so OR describe how participants were allocated to groups, and if allocation was not random, describe how covariates were controlled.*

Ecological, evolutionary & environmental sciences study design

All studies must disclose on these points even when the disclosure is negative.

Study description	*Briefly describe the study. For quantitative data include treatment factors and interactions, design structure (e.g. factorial, nested, hierarchical), nature and number of experimental units and replicates.*
Research sample	*Describe the research sample (e.g. a group of tagged Passer domesticus, all Stenocereus thurberi within Organ Pipe Cactus National Monument), and provide a rationale for the sample choice. When relevant, describe the organism taxa, source, sex, age range and any manipulations. State what population the sample is meant to represent when applicable. For studies involving existing datasets, describe the data and its source.*
Sampling strategy	*Note the sampling procedure. Describe the statistical methods that were used to predetermine sample size OR if no sample-size calculation was performed, describe how sample sizes were chosen and provide a rationale for why these sample sizes are sufficient.*
Data collection	*Describe the data collection procedure, including who recorded the data and how.*
Timing and spatial scale	*Indicate the start and stop dates of data collection, noting the frequency and periodicity of sampling and providing a rationale for these choices. If there is a gap between collection periods, state the dates for each sample cohort. Specify the spatial scale from which the data are taken*
Data exclusions	*If no data were excluded from the analyses, state so OR if data were excluded, describe the exclusions and the rationale behind them, indicating whether exclusion criteria were pre-established.*
Reproducibility	*Describe the measures taken to verify the reproducibility of experimental findings. For each experiment, note whether any attempts to repeat the experiment failed OR state that all attempts to repeat the experiment were successful.*
Randomization	*Describe how samples/organisms/participants were allocated into groups. If allocation was not random, describe how covariates were controlled. If this is not relevant to your study, explain why.*

Blinding

Describe the extent of blinding used during data acquisition and analysis. If blinding was not possible, describe why OR explain why blinding was not relevant to your study.

Did the study involve field work? ☐ Yes ☐ No

Field work, collection and transport

Field conditions

Describe the study conditions for field work, providing relevant parameters (e.g. temperature, rainfall).

Location

State the location of the sampling or experiment, providing relevant parameters (e.g. latitude and longitude, elevation, water depth).

Access & import/export

Describe the efforts you have made to access habitats and to collect and import/export your samples in a responsible manner and in compliance with local, national and international laws, noting any permits that were obtained (give the name of the issuing authority, the date of issue, and any identifying information).

Disturbance

Describe any disturbance caused by the study and how it was minimized.

Reporting for specific materials, systems and methods

We require information from authors about some types of materials, experimental systems and methods used in many studies. Here, indicate whether each material, system or method listed is relevant to your study. If you are not sure if a list item applies to your research, read the appropriate section before selecting a response.

Materials & experimental systems

n/a	Involved in the study	
☐	☒	Antibodies
☐	☒	Eukaryotic cell lines
☒	☐	Palaeontology and archaeology
☐	☒	Animals and other organisms
☐	☒	Clinical data
☒	☐	Dual use research of concern

Methods

n/a	Involved in the study	
☒	☐	ChIP-seq
☐	☒	Flow cytometry
☒	☐	MRI-based neuroimaging

Antibodies

Antibodies used

In vivo antibodies used:
Anti-PD-1 (10mg/kg i.p., Bio X Cell Cat# BE0146)
CTSS inhibitor (5mg/kg, i.p., APEx Bio)
Isotype control antibody (10mg/kg i.p., Bio X Cell Cat# BE0087)

Flow antibodies used:
Live/Dead Fixable Aqua stain (1:1000), CD11b-BV711 (M1/70) (1:200), CD68-APC/Cy7 (FA-11) (1:100), F4/80-PE/Dazzle (BM8) (1:200), I-A/I-E (M5/114.15.2) (1:200), Arginase 1 (A1exF5) (1:100). All antibodies were purchased from BioLegend, and the viability stain and Arginase 1 was purchased from ThermoFisher Scientific.

Validation

From BioXCell:
Exceptional Purity Our optimized proprietary antibody manufacturing method ensures an ultra-pure antibody solution without added proteins or chemicals. Each lot is QC tested for purity using SDS-PAGE.
Ultra-low Endotoxin Levels The level of endotoxin is QC tested for each lot. Our InVivoMAb products are at or below 2EU/mg and InVivoPlus products are at or below 1EU/mg. If endotoxin levels below 1EU/mg are required, please simply contact our technical support for details.
Pathogen Free Each lot of InVivoPlus product is screened for an exhaustive panel of murine pathogens. The results are detailed on product-specific datasheets, to help you adhere to IACUC and Animal Facility requirements.
Advanced Binding Validation We utilize a library of recombinant proteins and our bioassay expertise to validate that each lot of applicable InVivoPlus antibody binds strongly and specifically to its target antigen.
Low Protein Aggregation Our proprietary antibody manufacturing method ensures an antibody solution with very low levels of protein aggregation. Additionally, each lot of InVivoPlus product is QC tested for aggregate level and guaranteed to be below 5% of the total protein.
Matching Isotype Control Antibodies Bio X Cell carries a wide selection of non-binding isotype control antibodies. This takes the guesswork out of finding the correct control for your antibody.

From BioLegend:
Multiple Application Validation

Antibody clones are then tested in a variety of assays to see which applications they are suited for. As an example, clone 13A3-1 for phosphorylated STAT3 (Tyr705) demonstrated excellent performance in flow cytometry, western blot, and chromatin immunoprecipitation. Thus, the clone cross-validates itself by demonstrating functionality across orthogonal testing methods. Additionally, the biological induction of the phosphorylated state using IL-6 further validates the specificity of the antibody.
Flow Cytometry Reagents
Specificity testing of 1-3 target cell types with either single- or multi-color analysis (including positive and negative cell types).
Once specificity is confirmed, each new lot must perform with similar intensity to the in-date reference lot. Brightness (MFI) is evaluated from both positive and negative populations.
Each lot product is validated by QC testing with a series of titration dilutions.
The cross-reactivity of antibodies were verified here: https://www.biolegend.com/cross_reactivity

From ThermoFisher:
Thermo Fisher Scientific is committed to adopting higher validation standards for the Invitrogen antibody portfolio. We have implemented additional specificity tests to help ensure the highest confidence levels in our products. You can identify the products that have already undergone this testing with the Advanced Verification badge, shown above. This badge can be found in antibody search results and at the top of product webpages. The data supporting the Advanced Verification status can be found in the product specific data galleries. To learn more about our testing standards, please visit Invitrogen Antibody Validation.
Part 1. Target
specificity verification
Helps ensure the antibody will bind to the
correct target; our antibodies are being tested
using at least one of the following methods:
• Knockout
• Knockdown
• Independent antibody verification
• Cell treatment
• Relative expression
• Neutralization
• Peptide array
• SNAP-ChIP™ validation
• Immunoprecipitation/mass spectrometry
Part 2. Functional
application validation
These tests help ensure the antibody
works in particular application(s) of
interest, which may include (but are
not limited to):
• Western blotting
• Immunofluorescence imaging
• Flow cytometry
• ChIP
• Immunohistochemistry

Eukaryotic cell lines

Policy information about cell lines and Sex and Gender in Research

Cell line source(s)

2x105 MC38 (RRID:CVCL_B288) cells were transplanted into the flanks of mice. MC38 cells were generously provided by Dr. Andrew Sharabi.

Authentication

MC38 cells were not screened using STR profiled on site.

Mycoplasma contamination

Cell lines were not tested for mycoplasma contamination.

Commonly misidentified lines
(See ICLAC register)

MC38 is not a commonly misidentified cell line based on ICLAC version 11 Table 1.

Palaeontology and Archaeology

Specimen provenance

Provide provenance information for specimens and describe permits that were obtained for the work (including the name of the issuing authority, the date of issue, and any identifying information). Permits should encompass collection and, where applicable, export.

Specimen deposition

Indicate where the specimens have been deposited to permit free access by other researchers.

Dating methods

If new dates are provided, describe how they were obtained (e.g. collection, storage, sample pretreatment and measurement), where they were obtained (i.e. lab name), the calibration program and the protocol for quality assurance OR state that no new dates are provided.

☐ Tick this box to confirm that the raw and calibrated dates are available in the paper or in Supplementary Information.

Ethics oversight	*Identify the organization(s) that approved or provided guidance on the study protocol, OR state that no ethical approval or guidance was required and explain why not.*

Note that full information on the approval of the study protocol must also be provided in the manuscript.

Animals and other research organisms

Policy information about studies involving animals; ARRIVE guidelines recommended for reporting animal research, and Sex and Gender in Research

Laboratory animals	Wild-type female C57BL/6 (RRID:IMSR_JAX:000664) (4–6 weeks of age and weighing 16–18g) were purchased from The Jackson Laboratory. We exclusively used female mice in this study based on historical data and papers documenting the use of female C57BL/6 mice with the MC38 tumor model. Mice at Moores Cancer Center, UCSD are housed in micro-isolator and individually ventilated cages supplied with acidified water and fed 5053 Irradiated Picolab Rodent Diet 20 lab diet. More information regarding housing and handling of mice can be found in the methods section of the manuscript.
Wild animals	No wild animals were used in study.
Reporting on sex	MC38 cells were transplanted in female mice and treated with anti-PD-1.
Field-collected samples	No field-collected samples were used in study
Ethics oversight	All the animal studies were approved by the Institutional Animal Care and Use Committee (IACUC) of University of California, San Diego, with protocol ASP #S15195.

Note that full information on the approval of the study protocol must also be provided in the manuscript.

Clinical data

Policy information about clinical studies

All manuscripts should comply with the ICMJE guidelines for publication of clinical research and a completed CONSORT checklist must be included with all submissions.

Clinical trial registration	*Provide the trial registration number from ClinicalTrials.gov or an equivalent agency.*
Study protocol	*Note where the full trial protocol can be accessed OR if not available, explain why.*
Data collection	*Describe the settings and locales of data collection, noting the time periods of recruitment and data collection.*
Outcomes	*Describe how you pre-defined primary and secondary outcome measures and how you assessed these measures.*

Dual use research of concern

Policy information about dual use research of concern

Hazards

Could the accidental, deliberate or reckless misuse of agents or technologies generated in the work, or the application of information presented in the manuscript, pose a threat to:

No	Yes	
☐	☐	Public health
☐	☐	National security
☐	☐	Crops and/or livestock
☐	☐	Ecosystems
☐	☐	Any other significant area

Experiments of concern

Does the work involve any of these experiments of concern:

No	Yes	
☐	☐	Demonstrate how to render a vaccine ineffective
☐	☐	Confer resistance to therapeutically useful antibiotics or antiviral agents
☐	☐	Enhance the virulence of a pathogen or render a nonpathogen virulent
☐	☐	Increase transmissibility of a pathogen
☐	☐	Alter the host range of a pathogen
☐	☐	Enable evasion of diagnostic/detection modalities
☐	☐	Enable the weaponization of a biological agent or toxin
☐	☐	Any other potentially harmful combination of experiments and agents

ChIP-seq

Data deposition

☐ Confirm that both raw and final processed data have been deposited in a public database such as GEO.

☐ Confirm that you have deposited or provided access to graph files (e.g. BED files) for the called peaks.

Data access links<br>*May remain private before publication.*	*For "Initial submission" or "Revised version" documents, provide reviewer access links. For your "Final submission" document, provide a link to the deposited data.*
Files in database submission	*Provide a list of all files available in the database submission.*
Genome browser session<br>(e.g. UCSC)	*Provide a link to an anonymized genome browser session for "Initial submission" and "Revised version" documents only, to enable peer review. Write "no longer applicable" for "Final submission" documents.*

Methodology

Replicates	*Describe the experimental replicates, specifying number, type and replicate agreement.*
Sequencing depth	*Describe the sequencing depth for each experiment, providing the total number of reads, uniquely mapped reads, length of reads and whether they were paired- or single-end.*
Antibodies	*Describe the antibodies used for the ChIP-seq experiments; as applicable, provide supplier name, catalog number, clone name, and lot number.*
Peak calling parameters	*Specify the command line program and parameters used for read mapping and peak calling, including the ChIP, control and index files used.*
Data quality	*Describe the methods used to ensure data quality in full detail, including how many peaks are at FDR 5% and above 5-fold enrichment.*
Software	*Describe the software used to collect and analyze the ChIP-seq data. For custom code that has been deposited into a community repository, provide accession details.*

Flow Cytometry

Plots

Confirm that:

☒ The axis labels state the marker and fluorochrome used (e.g. CD4-FITC).

☒ The axis scales are clearly visible. Include numbers along axes only for bottom left plot of group (a 'group' is an analysis of identical markers).

☒ All plots are contour plots with outliers or pseudocolor plots.

☒ A numerical value for number of cells or percentage (with statistics) is provided.

Methodology

Sample preparation	Mice were euthanized and tumors were dissected and digested with Collagenase-D. After a single cell suspension was achieved, samples were processed for live/dead cell staining as well as fluorescence-labeled antibodies listed above.
Instrument	Novocyte Advanteon Flow Cytometer

Software	FlowJo™ v10.6
Cell population abundance	The abundance of live immune cells was characterized by LIVE/DEAD™ Fixable Aqua negative and CD11b+
Gating strategy	For all flow cytometry experiments, FMO samples were ran to determine placing of gates. The gating strategy is as follows: cells were gated for forward and side scatter, singlets were determined as the linear portion of forward scatter-area vs. forward scatter-height, live immune cells were gated as LIVE/DEAD™ Fixable Aqua negative and CD11b+, bulk macrophages were then gated from live lymphocytes as CD11b+ F4/80+, M1 macrophages were then characterized as MHCII+ F4/80+, M2 macrophages were then characterized as F4/80+ Arginase+

☒ Tick this box to confirm that a figure exemplifying the gating strategy is provided in the Supplementary Information.

Magnetic resonance imaging

Experimental design

Design type	*Indicate task or resting state; event-related or block design.*
Design specifications	*Specify the number of blocks, trials or experimental units per session and/or subject, and specify the length of each trial or block (if trials are blocked) and interval between trials.*
Behavioral performance measures	*State number and/or type of variables recorded (e.g. correct button press, response time) and what statistics were used to establish that the subjects were performing the task as expected (e.g. mean, range, and/or standard deviation across subjects).*

Acquisition

Imaging type(s)	*Specify: functional, structural, diffusion, perfusion.*
Field strength	*Specify in Tesla*
Sequence & imaging parameters	*Specify the pulse sequence type (gradient echo, spin echo, etc.), imaging type (EPI, spiral, etc.), field of view, matrix size, slice thickness, orientation and TE/TR/flip angle.*
Area of acquisition	*State whether a whole brain scan was used OR define the area of acquisition, describing how the region was determined.*

Diffusion MRI ☐ Used ☐ Not used

Preprocessing

Preprocessing software	*Provide detail on software version and revision number and on specific parameters (model/functions, brain extraction, segmentation, smoothing kernel size, etc.).*
Normalization	*If data were normalized/standardized, describe the approach(es): specify linear or non-linear and define image types used for transformation OR indicate that data were not normalized and explain rationale for lack of normalization.*
Normalization template	*Describe the template used for normalization/transformation, specifying subject space or group standardized space (e.g. original Talairach, MNI305, ICBM152) OR indicate that the data were not normalized.*
Noise and artifact removal	*Describe your procedure(s) for artifact and structured noise removal, specifying motion parameters, tissue signals and physiological signals (heart rate, respiration).*
Volume censoring	*Define your software and/or method and criteria for volume censoring, and state the extent of such censoring.*

Statistical modeling & inference

Model type and settings	*Specify type (mass univariate, multivariate, RSA, predictive, etc.) and describe essential details of the model at the first and second levels (e.g. fixed, random or mixed effects; drift or auto-correlation).*
Effect(s) tested	*Define precise effect in terms of the task or stimulus conditions instead of psychological concepts and indicate whether ANOVA or factorial designs were used.*

Specify type of analysis: ☐ Whole brain ☐ ROI-based ☐ Both

Statistic type for inference (See Eklund et al. 2016)	*Specify voxel-wise or cluster-wise and report all relevant parameters for cluster-wise methods.*
Correction	*Describe the type of correction and how it is obtained for multiple comparisons (e.g. FWE, FDR, permutation or Monte Carlo).*

Models & analysis

n/a	Involved in the study
☐	☐ Functional and/or effective connectivity
☐	☐ Graph analysis
☐	☐ Multivariate modeling or predictive analysis

Functional and/or effective connectivity	*Report the measures of dependence used and the model details (e.g. Pearson correlation, partial correlation, mutual information).*
Graph analysis	*Report the dependent variable and connectivity measure, specifying weighted graph or binarized graph, subject- or group-level, and the global and/or node summaries used (e.g. clustering coefficient, efficiency, etc.).*
Multivariate modeling and predictive analysis	*Specify independent variables, features extraction and dimension reduction, model, training and evaluation metrics.*
